# 2555. Metronidazole Dosing in Patients with and without Obesity Undergoing Colorectal Surgery: Should body size or composition be a factor?

**DOI:** 10.1093/ofid/ofad500.2172

**Published:** 2023-11-27

**Authors:** Manjunath P Pai, Aleksas Matvekas, Krishani Rajanayake, Theresa Carrier, Allison Nguyen, Brian Ross, June Sullivan, Radin Alikhani, Mohamed Abdelnabi, Scott Regenbogen, John Byrn, Grace Su, Stewart Wang

**Affiliations:** University of Michigan, Ann Arbor, Michigan; University of Michigan, College of Pharmacy, Ann Arbor, Michigan; University of Michigan, College of Pharmacy, Ann Arbor, Michigan; University of Michigan, College of Pharmacy, Ann Arbor, Michigan; University of Michigan, College of Pharmacy, Ann Arbor, Michigan; University of Michigan, Michigan Medicine, Department of Surgery, Ann Arbor, Michigan; University of Michigan, Michigan Medicine, Department of Surgery, Ann Arbor, Michigan; University of Michigan, College of Pharmacy, Ann Arbor, Michigan; University of Michigan, College of Pharmacy, Ann Arbor, Michigan; University of Michigan, Michigan Medicine, Department of Surgery, Ann Arbor, Michigan; University of Michigan, Michigan Medicine, Department of Surgery, Ann Arbor, Michigan; University of Michigan, Michigan Medicine, Department of Surgery, Ann Arbor, Michigan; University of Michigan, Michigan Medicine, Department of Surgery, Ann Arbor, Michigan

## Abstract

**Background:**

Metronidazole (MET) is administered on a fixed-dose basis irrespective of body size in adults but studies suggest that dose adjustment may be needed (PMID: 33969405). This study evaluated plasma and fat tissue pharmacokinetics (PK) of MET in patients undergoing elective colorectal surgery.

**Table 1.**
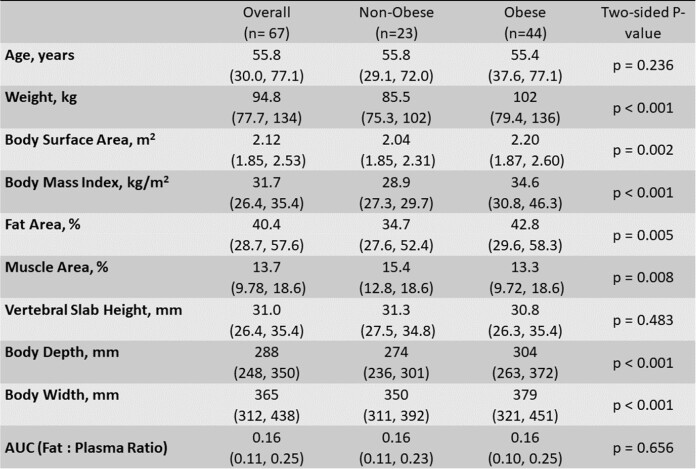
Patient demographics, body size, morphomic and pharmacokinetic values by obese group. Data reported as median (5th, 95th percentile) values

**Figure 1.**
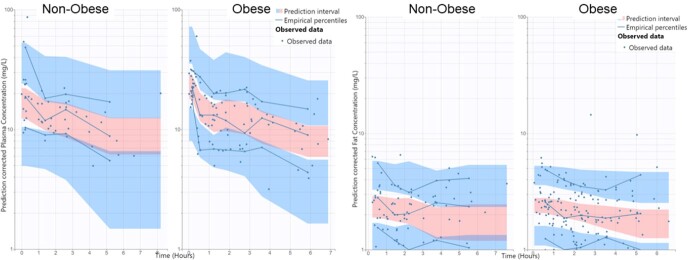
Visual predictive check illustrating observed and model predicted metronidazole concentrations in plasma and fat tissue samples stratified by patients with and with out obesity

**Methods:**

Adult patients (age ≥ 18 years) undergoing elective colorectal procedures with a preoperative CT scan available were prospectively enrolled. MET was administered as standard of care (500 mg) intravenously 15-60 prior to incision with blood sampling performed at the end of infusion, before incision, and at surgical closure. Fat tissue samples were collected at incision and closure. Samples were assayed by an LC-MS/MS method. Population PK analyses were performed by co-modeling plasma and fat MET concentrations and comparing weight, body surface area (BSA), morphomics, and conventional patient demographics as covariates. Morphomics are body dimensions and composition (e.g. muscle and fat areas) parameters generated using CT scan data.

**Results:**

Data were acquired from 67 patients (n=36 male) with comparisons between groups reported in Table 1. A 1-compartment model adequately characterized plasma MET profiles with a mean (CV) volume of distribution (V) and clearance (CL) of 24.4 (62.5%) L and 4.04 (34.2%) L/h. A two-compartment structure with transfer rate constants, with vertebral height (compared to weight or BSA) as a covariate of V and age as a covariate of CL provided the best co-model fit for plasma and fat tissue (Figure 1). The median [5^th^, 95^th^ percentile] sampling period was 3.3 [1.6, 6.4] hours with an AUC_τ_ of 44.0 [22.0, 83.4] h*mg/L and 6.79 [3.11, 13.6] h*mg/L in plasma and fat tissue. No significant difference was observed in plasma or fat exposures or their ratio as a surrogate for tissue penetration between obese and non-obese patients. Simulations suggest a lower probability (≤62.6%) of achieving an AUC_∞_/MIC of 70 in adults under the age of 50 years.

**Conclusion:**

Plasma and fat tissue exposures of MET are similar in obese and non-obese patients undergoing elective colorectal surgeries. Age may be a more relevant factor than body size or composition for metronidazole dose adjustment.

**Disclosures:**

**Stewart Wang, MD, PhD**, Applied Morphomics, Inc.: patent for analytic morphomics|Applied Morphomics, Inc.: Ownership Interest|mBioHealth: Ownership Interest|Surgical Risk Management: Ownership Interest

